# Toward a Gecko-Inspired, Climbing Soft Robot

**DOI:** 10.3389/fnbot.2019.00106

**Published:** 2019-12-19

**Authors:** Lars Schiller, Arthur Seibel, Josef Schlattmann

**Affiliations:** Workgroup on System Technologies and Engineering Design Methodology, Hamburg University of Technology, Hamburg, Germany

**Keywords:** mobile soft robots, fast pneu-nets, apriltags, gecko-inspired robot, climbing robot

## Abstract

In this paper, we present a gecko-inspired soft robot that is able to climb inclined, flat surfaces. By changing the design of the previous version, the energy consumption of the robot could be reduced, and at the same time, its ability to climb and its speed of movement could be increased. As a result, the new prototype consumes only about a third of the energy of the previous version and manages to climb slopes of up to 84°. In the horizontal plane, its velocity could be increased from 2 to 6 cm/s. We also provide a detailed analysis of the robot's straight gait.

## 1. Introduction

In the last decade, soft robotics has become an established field in the robotics sciences, and is still growing rapidly. This discipline utilizes the properties of soft materials and structures for developing new types of machines showing a compliance similar to that of living organisms (Majidi, 2014). Examples of biological models include worms, caterpillars, and cephalopods (Kim et al., 2013).

**Figure 1:**
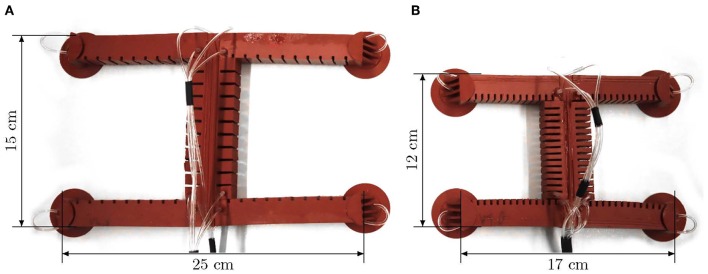
Current prototypes of the gecko-inspired soft robot. **(A)** Large prototype, **(B)** small prototype.

Typically, soft robots are designed either for locomotion or for grasping and manipulation (Rus and Tolley, 2015). In the context of locomotion, typical principles are crawling, walking, running, jumping, flying, and swimming (Calisti et al., 2017). The zoo of soft robots includes representatives of all these principles. Climbing—a combination of locomotion and adhesion (Chu et al., 2010)—, however, is largely unexplored, besides a few exceptions (Gu et al., 2018; Tang et al., 2018; Qin et al., 2019). A great advantage of flexible machines is that they can hardly endanger themselves or their environment due to their softness. This raises the question why there are no universal, climbing soft robots, as they can easily survive a fall—Universal in the sense that they can move in any direction. In Seibel and Schiller (2018), we therefore introduced a soft robot that is specifically designed for climbing. Figure 1A shows a slightly modified version of the robot presented therein. Its design is based on the use of fast pneu-net bending actuators (Mosadegh et al., 2014) as the primary element and its locomotion is inspired by the gecko (Autumn et al., 2006). The attachment of the soft robot to the ground during gait is realized by suction cups as feet. The robot presented therein is able to climb surfaces up to 50° inclination.

However, the robot is linked to an external pressure source by supply tubes. In order to enable autonomous movements, it would have to carry its pressure source with it. Therefore, its movements should be as energy efficient as possible so that a potentially more lightweight pressure source can be used. In this paper, we investigate how the energy consumption of the robot can be reduced by constructive measures. This is an important step in the direction of mobile, untethered soft robotics.

## 2. New Prototype of the Robot

The new prototype of our gecko-inspired soft robot is designed in terms of a reduction of its pneumatic energy consumption. This is mainly achieved by downscaling the previous version. Therefore, the new prototype is denoted as small version and the previous one as large version. In the following, the robot's redesign process is described in detail.

### 2.1. Energy Consumption

The energy consumption of a pneumatic system is strongly related to its air demand, which can be calculated by multiplying the volume of the pneumatic system by the applied pressure. For a qualitative comparison, the energy consumption is considered equal to the air demand, as the used control unit is identical for both prototypes, and therefore, other factors affect both versions in the same fashion. In order to obtain a meaningful value for the energy consumption *E* of the robot, the air demand is set in relation to the shift in position:

(1) $$\begin{array}{l} \frac{E}{\Delta x}=\frac{n_{\text{cyc}}\sum_{i}^{N}p_{i}V_{i}n_{i}}{x_{\text{end}}-x_{\text{start}}}\;. \end{array}$$

Here, *p*_*i*_ describes the applied pressure in the *i*th actuator (from a total of *N* actuators) with the volume *V*_*i*_, and *n*_*i*_ is the number of actuations of this actuator within a gait cycle. Furthermore, *n*_cyc_ describes the number of cycles necessary to move from the start position *x*_start_ to the end position *x*_end_. With a constant distance Δ*x* traveled, there are three ways to reduce the energy consumption:

- Maximize the shift in position per cycle and thus minimize the required number of cycles *n*_cyc_,
- Reduce the required pressures *p*_*i*_ for the actuators while keeping the shift in position per cycle constant.
- Reduce the inner volumes *V*_*i*_ of the actuators.

The shift in position per cycle can be approximately estimated with one body length, as we will see in **Figure 6** and Equation (5). In order to increase the shift in position, all six actuators of the robot have to be increased in size, which only has an effect by a factor of one on the shift in position, but by a factor of six on the air demand. Therefore, it is more promising to reduce the volume and the required pressure than to increase the shift in position per cycle.

### 2.2. Design Parameters and Realization of the Small Prototype

The most effective way to reduce the internal volume of a fast pneu-net actuator is to increase the number of chambers. This not only reduces the inner volume, but also increases the self-reinforcing effect of the actuator and thus also reduces the required pressure, as experimentally studied in Mosadegh et al. (2014) (and approved by own experiments documented in the Supplementary Data). From simulations in Polygerinos et al. (2013), it is also known that thinner walls lead to less required pressure and increased force output. Additionally, increasing the chamber height increases the force output. From these findings, it can be concluded that an actuator with many chambers, thin inner walls, and a big chamber height is desirable for best performance.

The design of the large prototype is based on the actuator dimensions from the Soft Robotics Toolkit (Website, 2019). In order to reduce the volume of the actuator, only the overall length ℓ_act_ and the number of chambers *n*_ch_ should be adjusted so that the fittings to the suction cups are not changed. The width *w*_ch_ and height *h*_ch_ of the chambers should therefore remain constant. The manufacturing process (3D printed molds, manual injection molding with Elastosil) makes it difficult to achieve wall thicknesses of less than 1 mm. Therefore, the inner wall thickness *t*_w,i_ is not varied either. The total length chosen is ℓ_act_ = 76.5 mm. Own experiments have shown that a leg of this length has an advantageous stiffness to be integrated into the robot. As many chambers as possible are arranged on this length, which is *n*_ch_ = 15. The limiting factor here is the fragility of the 3D-printed molds. The inner volume *V* of an actuator can now be calculated using the following equation:

(2) $$\begin{array}{l} V=n_{\text{ch}}\left(h_{\text{ch}}\cdot w_{\text{ch}}\cdot \ell_{\text{ch}}\right)+\left(n_{\text{ch}}-1\right)\left(h_{\text{air}}\cdot w_{\text{air}}\cdot \ell_{\text{air}}\right), \end{array}$$

where *h*_air_, *w*_air_, and ℓ_air_ describe the height, width, and length of the air channel, respectively. It should be noted that this equation only describes the inner volume of an actuator at rest. If the actuator is actuated, the volume increases. However, for a qualitative comparison between the two versions, this approximation should be sufficient. Table 1 shows all necessary parameters for the design of both the large and the small version.

**Table 1:** Design parameters of the large and the small prototype of the gecko-inspired soft robot.

**Design parameters**		**Large prototype**	**Small prototype**	
Number of chambers	*n*_ch_	11	15	1
Actuator length	ℓ_act_	112	76.5	mm
Bottom layer height	*h*_bot_	5	5	mm
Outer wall thickness	*t*_w,o_	2	2	mm
Inner wall thickness	*t*_w,i_	1	1	mm
Chamber height	*h*_ch_	15	15	mm
Chamber width	*w*_ch_	11	11	mm
Chamber length	*l*_ch_	6	1.43	mm
Air channel height	*h*_air_	2	2	mm
Air channel width	*w*_air_	2	2	mm
Air channel length	*l*_air_	4	4	mm
Inner volume	*V*	0.01105	0.0038	m^3^

**Figure 2:**
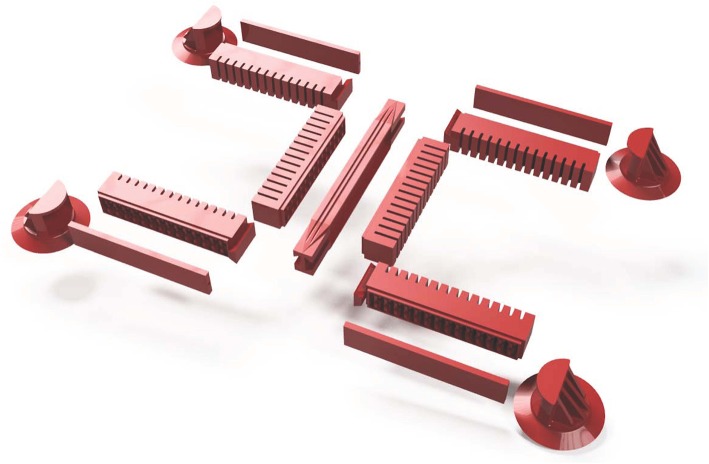
Explosion view of the small prototype of the gecko-inspired soft robot.

The design of the small prototype is basically the same as described in Seibel and Schiller (2018). In addition to the size variation, two more details have changed. The supply tubes for the front feet and legs no longer lie in the torso's middle layer, but outside the robot. This allows the middle layer of the torso to be designed with minimal thickness, and the torso is more flexible overall, requiring less pressure to deform it. In addition, there is a dovetail at the ends of the legs and a groove at the ends of the torso to make the process of joining the torso and legs more precise and easier. Figure 2 shows an exploded view of the small prototype. All parts of the robot have to be manufactured individually and joined afterwards. A photograph of the small prototype is shown in Figure 1B. In order to be able to make a meaningful comparison between the two versions, the large prototype of the robot was also manufactured with external supply tubes and dovetail joints (see Figure 1A).

## 3. Experiments

**Figure 3:**
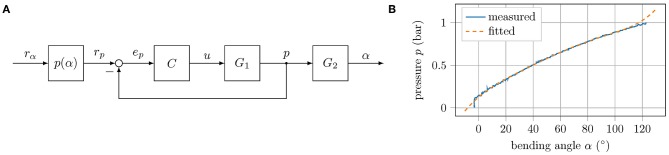
Control scheme: **(A)** Block diagram of the control loop for a single actuator. The block *p*(α) maps angle to pressure coordinates, *C* is the implemented PID controller, *G*_1_ describes the dynamics of the proportional valve, and *G*_2_ represents the dynamics of the tube and actuator. **(B)** Measured and fitted relation between bending angle and applied pressure in the horizontal plane of the left front leg of the small version.

**Figure 4:**
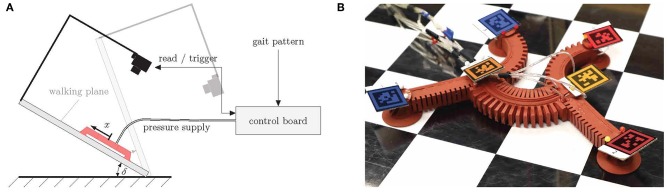
Experimental setting. **(A)** Principal sketch, **(B)** apriltags attached to the robot's feet and torso's ends. The individual tags are indicated by different colors (red—front left, dark red—front right, orange—front center, dark orange—rear center, blue—rear left, dark blue—rear right).

The robot consists of fast pneu-net bending actuators that take pressure as input. In most cases, however, the robot should take on a certain pose, that is, its limbs should have particular bending angles. For this reason, the angular input must be converted into pressure references. Figure 3 illustrates the control scheme for a single actuator. In A, the block diagram is shown. The angular reference *r*_α_ is converted into a reference pressure *r*_*p*_ by a mapping function *p*(α), depicted in B, which must be determined by an experiment. In comparison with the actual pressure, the PID controller *C* is fed, which in return generates the control signal *u* for the proportional directional valve *G*_1_. The pressure *p* is measured by a digital pressure sensor at the valve outlet. In the quasi-static case, this is the pressure inside the actuator *G*_2_, whose output is the bending angle α. A system of six of these channels connected in parallel to a compressor is required to operate all limbs of the robot. Control of the suction cups is realized by direct acting solenoid valves that are connected in parallel to a vacuum pump. All components required for control are combined in a compact, universal control board. In order to measure the bending angles of the robot, a camera is mounted above the walking plane (acrylic glass plate with an adjustable slope), as shown in Figure 4A. Apriltags (Wang and Olson, 2016) are attached to the feet and the torso ends of the robot (see Figure 4B). The position and orientation of the individual tags (and thus the position and orientation of the robot limbs) can now be detected in the camera images. In this way, the bending angles of the individual limbs can be calculated as follows:

(3) $$\hat{\alpha }(r_{1},r_{2})=\{\begin{array}{l} \varphi_{1}=\text{ atan}2(r_{y,1},r_{x,1})\\ {\tilde{r}}_{2}=R(-\varphi_{1})r_{2}\\ \hat{\alpha }\;=\text{ atan}2({\tilde{r}}_{y,2},{\tilde{r}}_{x,2}) \end{array},$$

where ***r***_1_ describes the apriltag's orientation of one and ***r***_2_ of the other end of a limb. Furthermore, ***R*** is the two-dimensional rotation matrix. The orientation angle of the robot ε is defined as the angle between the *x*-axis and the vector from the rear to the front end of the torso:

(4) $$\begin{array}{l} \varepsilon =\hat{\alpha }\left(e_{x},p_{1}-p_{4}\right). \end{array}$$

Here, *e*_*x*_ describes the unit vector in *x*-direction, ***p***_1_ the position of the front end, and ***p***_4_ the position of the rear end of the torso. All of the following experiments were repeated at least five times. In the following graphs, solid curves represent the mean value and the standard deviation is represented by an area, unless stated otherwise. Note that the measured bending angles are only used for analysis. The locomotion of the robot itself is controlled only by the pressure and the corresponding mapping function. The control scheme is intentionally kept as simple as possible in order to have as few dependencies on measurement systems as necessary and thus be able to run outside laboratory conditions.

### 3.1. Determining the Transient Time

**Figure 5:**
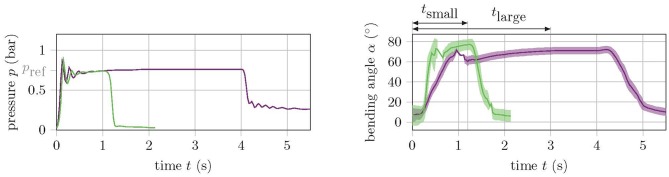
Step response of a small (green) and a large (purple) soft actuator for a reference pressure of *p*_ref_ = 0.75 bar. The left graph shows the pressure and the right graph shows the angle response.

The diameter and length of the supply tubes as well as the control hardware and software are identical for both versions. However, since the inner volume of the actuators is different, the small version requires comparatively less air to be transported through the tubes. This requires correspondingly less time and reduces the robot's movement phase, and thus also the cycle time. Figure 5 shows the step response for a reference pressure of *p*_ref_ = 0.75 bar of a small (green) and a large (purple) actuator. The bending angle of the small version reaches its equilibrium position after about one second, while the transient time of the large version lasts ~3 s.

### 3.2. Experiments on the Horizontal Plane

**Figure 6:**
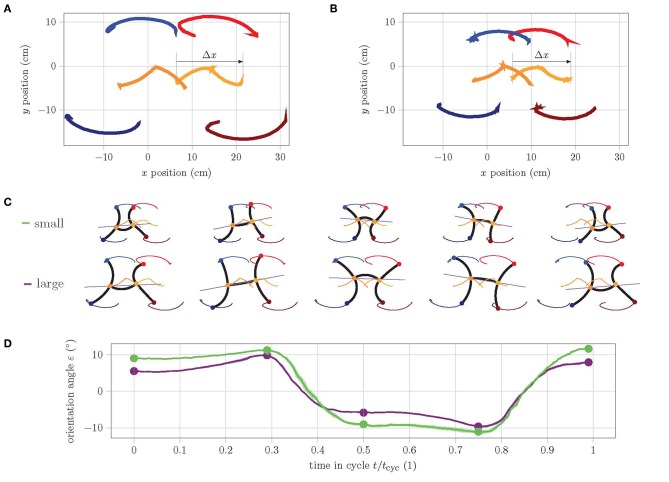
Analysis of the track of feet during one cycle of straight gait in the horizontal plane. **(A)** Track of large prototype, **(B)** track of small prototype. **(C)** Single frames show the abstraction of pose at the extreme points as well as in the start, middle, and end position during cycle. **(D)** Graph of orientation angle ε during cycle of small (green) and large (purple) prototype.

In a first experiment, the behavior of the two robot versions in the horizontal plane is analyzed. The aim of this experiment is to understand and compare the motion within a cycle. In order to obtain measurement data for the entire cycle, the gait pattern is slowed down since the camera images are smeared when the movements are too fast and the tags can no longer be detected. Figure 6 shows the track of the tags placed on the large (Figure 6A) and small (Figure 6B) prototype during a cycle. Apart from the dimensions, both robots have a very similar motion quality. However, the overlaps of the track of the torso ends (orange and dark orange) as well as the left feet (red and blue) are much larger in the small version. In fact, the small version has a much better ratio of shift in position within a cycle Δ*x* to body length ℓ_bl_ = ℓ_act_ + 2(*h*_ch_ + *h*_bot_ + *t*_w,o_):

(5) $$\begin{array}{l} \frac{\Delta x_{\text{small}}}{\ell_{\text{bl},\text{small}}}\approx \frac{12.5}{12.05}=1.04>0.96=\frac{15}{15.6}\approx \frac{\Delta x_{\text{large}}}{\ell_{\text{bl},\text{large}}}\;. \end{array}$$

This is mainly due to the fact that the torso of the small version has a better bending performance, as can be seen in the single frames of start, middle, and end pose in Figure 6C. Furthermore, the *y*-symmetry of the track of the torso ends is notable. Apparently, the rear torso end in the second half of the cycle qualitatively performs the same movement as the front torso end in the first half. Similarly, the movement of the rear torso end in the first half of the cycle is complementary to the movement of the front end in the second half. This necessarily results in a change of the orientation angle ε within a cycle. Figure 6D shows the history of ε during one cycle of straight gait. It can clearly be seen that the change in orientation within a cycle for the small version is larger than for the large version. The linear model for straight gait from Seibel and Schiller (2018) predicts the maximum displacement of the fixed feet for the poses at 25% and 75% of cycle time (i.e., approximately at the points of maximum orientation). This reveals how the problem of the model is solved in reality: the robot changes its orientation. The larger change of the small version is explained by the fact that the ratio of body length to leg length λ = ℓ_bl_/ℓ_act_ is comparatively larger:

(6) $$\begin{array}{l} \lambda_{\text{small}}=1.58>1.40=\lambda_{\text{large}}\;. \end{array}$$

For a larger ratio λ, the linear model predicts a larger displacement of the fixed feet. Since a displacement of the fixed feet is physically not possible, this theoretically larger displacement results in a larger change of orientation. This effect can be seen very well in the selected poses in Figure 6C.

### 3.3. Experiments on the Inclined Plane

**Figure 7:**
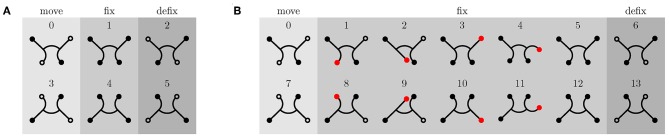
Gait patterns for straight movement of the robot. Fixed feet are indicated by filled circles and unfixed feet by unfilled circles. **(A)** Gait pattern for inclination angles δ < 70°. **(B)** Climbing pattern for high inclinations (δ ≥ 70°). Vacuum is applied to red and black filled feet. Black filled feet are fixed to the ground, whereas red feet do not necessarily have to be. In order to secure the fixation, the foot to be fixed is swinged back and forth once.

The gait pattern for the robot's straight gait consists of a total of six phases, of which two are complementary. This results in three categorical phases: movement phase, fixation phase, and release phase. Figure 7A shows the poses the robot should take within these phases. Based on the determination of the transient time in the previous section, the duration of the movement phase of the large version is set to three seconds and that of the small version to one second. The fixation phase lasts 0.1 seconds and the release phase also 0.1 seconds. Both are the same for both versions of the robot.

Within these phases, the reference bending angle for all limbs is either 0° or 90°. This angle corresponds in the horizontal plane and without external load to a certain pressure: the reference pressure (compare to Figure 3B). In the horizontal plane, the gravity force is perpendicular to the direction of movement. As the inclination angle δ increases, however, the gravity acts increasingly against the direction of movement of the robot, and therefore, the reference pressure must be adjusted in order to correspond to a bending angle of 90°. This means that the mapping function *p*(α) of each actuator must be recalibrated for each inclination.

**Figure 8:**
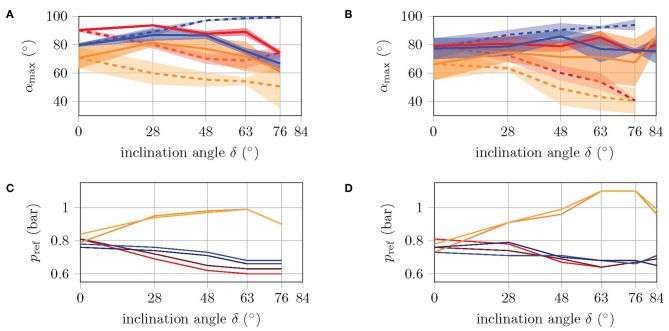
Maximum bending angle **(A,B)** and corresponding reference pressures **(C,D)** of front legs (red), rear legs (blue), and torso (orange) during a cycle. By applying the same pressure reference for different inclination angles (i.e., different loads), the dashed curves are obtained. The solid curves are obtained from the recalibrated pressure references, such that the bending angles match 90° as good as possible. Left diagrams are for large prototype and right diagrams for small prototype.

In the following experiment, the velocity and energy consumption of the two robot versions are determined for different inclination angles δ. First, the same reference pressures are used for all the inclinations, without recalibrating the mapping function. Then, the reference pressures are adjusted according to the inclination angle, so that the bending angles in the extreme positions correspond as closely as possible to 90°. Figures 8A,B show how the maximum bending angles of the front legs and the torso decrease with increasing inclination, while the bending angles of the rear feet increase, when using the same pressure references (dashed curve). This is, however, to be expected since the front legs and torso need to operate against gravity, while the actuation of the rear legs is supported by gravity.

The solid curves in Figures 8A,B show the maximum bending angles for the recalibrated reference pressures. In Figures 8C,D, the values for the reference pressures are shown for each inclination. Especially the torso is more pressurized, while the reference pressures in the legs are slightly decreased. This indicates that the shift in position comes mainly from the torso, which is consistent with the finding from Fischer and Witte (2007) of the torso as the main organ of locomotion. The high standard deviation is mainly due to the fact that in some cases, the feet are not fixed immediately during the fixation phase, but rather during the subsequent movement phase. This results in a foot position that does not correspond to the gait model, and may not allow the corresponding limb to bend by 90°.

However, as the angle of inclination increases, not only does the load increase (due to the counteracting gravity force), but the normal force, which pushes the feet against the walking plane, decreases to the same extent. Due to the small normal force at high slopes (δ > 70°), the sealing lip of the suction cup may not be in contact with the walking plane over the entire circumference, which may results in a gap. In this case, the suction cup cannot grip despite vacuum being applied. Therefore, a new gait pattern for high inclinations is introduced, as depicted in Figure 7B. The movement and release phases remain identical, but the fixation phase is extended by four additional poses. Before the two supporting feet are released, the potentially unfixed rear leg is first swung forwards and backwards once, in the expectation that the corresponding foot will be fixed during this motion. Afterwards, the same happens to the potentially unfixed front leg. Then, both supporting feet are released and the next movement phase begins. In the Supplementary Video (from 1:34 min on), it can be seen that the foot to be fixed is sucked into a random location within the swinging motion. With additional pressure sensors, it would be possible to detect whether a foot is fixed or not. Through this feedback, the execution of the swing phase of an already fixed leg can be avoided or the swing phase could be repeated if the first run did not have the desired effect. But as the experiments show, the one-time execution of the swing phase is sufficient, and the time saving by avoiding unnecessary swing phases is not particularly large. Therefore, this hardware upgrade has been omitted so far.

**Figure 9:**
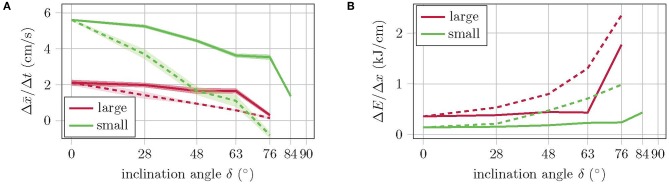
**(A)** Walking performance and **(B)** energy consumption for various inclination angles. Dashed curves show the values for constant and solid curves for recalibrated pressure references.

The experiment with constant reference pressures shows that the velocity linearly decreases with increasing inclination angle, while the energy consumption increases, see Figure 9. The small version has about twice the velocity with half the energy consumption. Both versions manage to climb slopes of up to 63° without falling. At a slope of 76°, the large version does not come from the spot, while the small one even slides backwards. In the experiment with recalibrated reference pressures, the velocity and energy consumption of the large version remain almost constant up to an inclination angle of 63°. This is because the number of cycles required remains the same, while the applied pressure increases only slightly [refer Equation (1)]. With the climbing pattern for high inclinations (see Figure 7B), the large version manages to climb inclinations of up to 76°. Higher inclination angles, however, are not possible with this version as the torso cannot reach the corresponding bending angles without running into danger of bursting due to the required applied pressure. The velocity of the small robot version with recalibrated reference pressures is significantly higher compared to the performance with constant pressure references, but decreases with increasing inclination. Energy consumption is also comparatively lower. The small version even manages to master inclinations of up to 84° and is therefore very close to the goal of climbing a vertical wall. A further increase of the inclination, however, does fail here due to a lack of fixation. The normal force is simply too small to bring the suction cups close enough to the walking plane to allow them to suck. Since the robot cannot actively push its feet against the plane of movement, no further variation of the gait pattern will help at this point. A mechanism is needed that pushes the feet against the plane.

The complete data set of the experiments on the inclined plane is provided in the Supplementary Data.

## 4. Conclusion

The goal of this paper was to develop a new version of the gecko-inspired soft robot from Seibel and Schiller (2018) that consumes less energy in order to take another step in the direction of an untethered, soft climbing robot. By reducing the volume and increasing the self-reinforcing effect of the bending actuators, not only this goal could be achieved, but also the velocity of the robot was significantly increased (by 300%), as well as its ability to climb. Table 2 summarizes the results of this study. The large version has major difficulties in reaching the reference pose at all when climbing high inclinations, that is, the actuators do not provide enough force to bend to the desired angle under high load. Increasing the reference pressure would exceed the actuator's capability and cause it to burst. For the small version, however, climbing is not limited by a lack of force, but by a lack of fixation. Reaching the reference pose is no challenge for this version, even under high load. In order to improve the fixation, only a mechanism is needed that allows the robot to actively push its feet to the plane of movement. With such a mechanism, the robot could easily climb vertical walls.

**Table 2:** Comparison of the large and the small prototype of the gecko-inspired soft robot.

	**Large prototype**	**Small prototype**	
Speed on horizontal plane	2 (0.13)	6 (0.5)	cm/s (ℓ_bl_/s)
Energy consumption on horizontal plane	0.36	0.13	kJ/cm
Total weight	200	150	g
Average applied pressure	0.80	0.76	bar
Ability to climb	76	84	°

Compared to other robots, this robot can move extremely fast. Qin et al. (2019) summarize the speeds of different soft crawling robots and introduces a novel robot with special emphasis on rapid locomotion, since its speed is much higher than of previous soft robots. Without a payload, this robot moves at a speed of about 0.1 ℓ_bl_/s, while our robot can run five times the speed related to its body length.

In order to free the robot from its umbilical cord, a suitable on-board pressure and vacuum source must be found. Even though research is ongoing (Adami and Seibel, 2019), this task is still open. In any case, reducing energy consumption to 36% compared to the previous version brings this goal closer.

## Data Availability Statement

The datasets exp_incl_plane and exp_slow_track for this study can be found in the collection Research Data TUHH [https://doi.org/10.15480/336.2519].

## Author Contributions

LS and AS initiated the study and wrote the manuscript. LS redesigned the robot, performed the experiments, and discussed the results. LS, AS, and JS revised the manuscript. AS and JS supervised the project.

### Conflict of Interest

The authors declare that the research was conducted in the absence of any commercial or financial relationships that could be construed as a potential conflict of interest.

## Supplementary Material

Supplementary Video The video supporting the conclusions of the experiments is included as additional file.

Supplementary Data A pdf containing a experimental design parameter study and all relevant data measured during the experiments on the inclined plane is also included as additional file.
