# Supplementary material for: Toward a Gecko-Inspired, Climbing Soft Robot
Source: Front Neurorobot. 2019 Dec 19;13:106. doi: 10.3389/fnbot.2019.00106 (PMC6951426; doi:10.3389/fnbot.2019.00106)
Supplement: Supplementary Data — A pdf containing a experimental design parameter study and all relevant data measured during the experiments on the inclined plane is also included as additional file. [file Data-Sheet_1.PDF]

frontiers

Towards a gecko-inspired, climbing soft robot Supporting Information

Lars Schiller [1,*], Arthur Seibel [1] and Josef Schlattmann [1]

[1] *Workgroup on System Technologies and Engineering Design Methodology, Hamburg University of Technology, 21073 Hamburg, Germany*

Correspondence*:
Lars Schiller
lars.schiller@tuhh.de

1 EXPERIMENTAL DESIGN PARAMETER STUDY

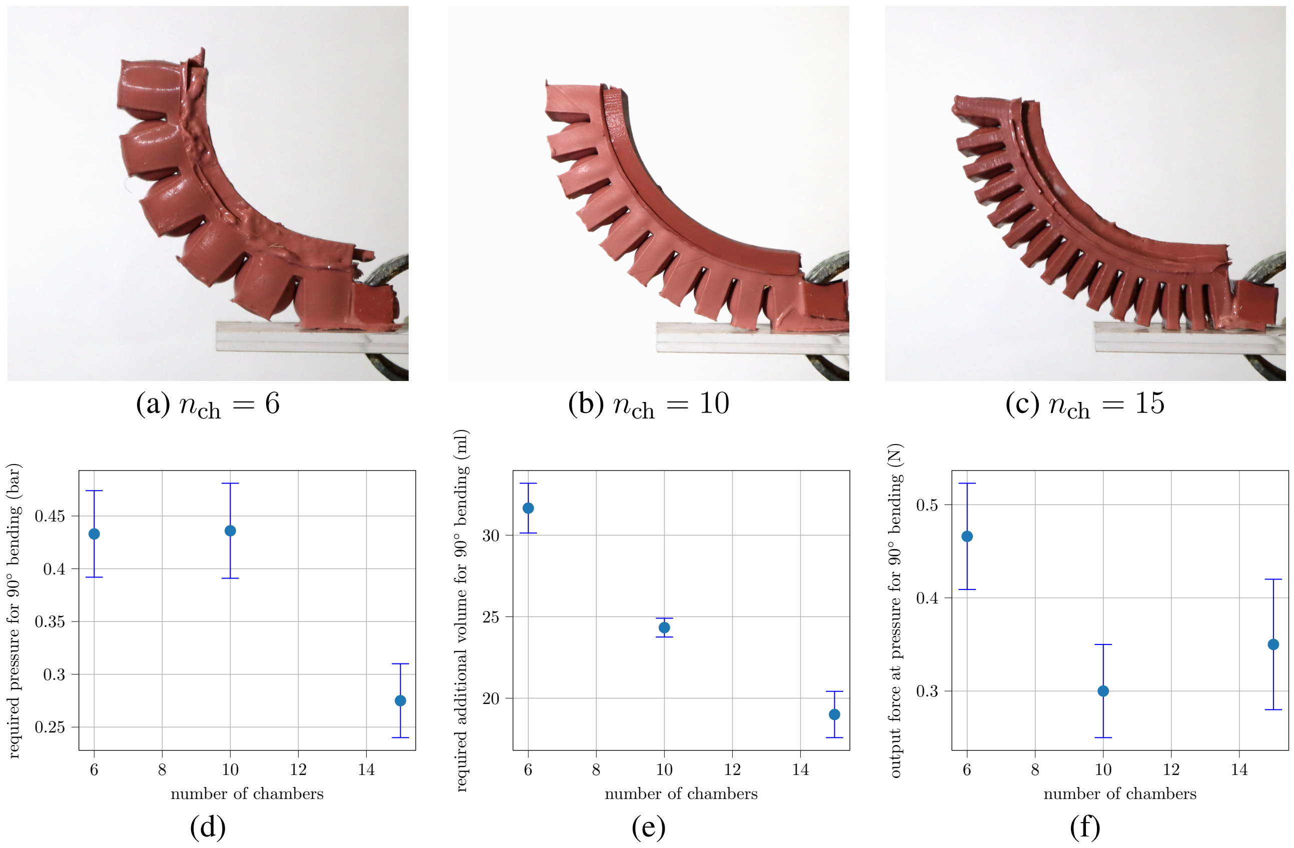

Figure 1. Experimental results for different numbers of chambers. The overall length and height are constant in this experiment. A total of three different numbers of chambers were tested, with at least three actuators of each type manufactured and tested. The subfigures (a)–(c) show tested actuators. (d) Shows the pressure needed to bend the actuator by $90°$ against gravity. (e) Shows the additional air volume injected into the actuator to reach the $90°$ bending angle (derived from the piston displacement of a syringe). (f) Shows the force that the actuator exerts on a force sensor that fixes it in the rest position when it is subjected to the pressure determined in (d).

2 EXPERIMENTS ON THE INCLINED PLANE

The following graphs show all relevant collected data during the presented study. All of the experiments were repeated at least five times. In the following graphs that contain a time axis, solid curves represent the mean value and the standard deviation is represented by an area. Figure 2 and 3 are exceptions of this rule, since these graphs do not contain a time axis. Here, the mean value of a measured quantity is indicated again by a solid curve, but the standard deviation is represented by an ellipse, where the radius in the x-direction corresponds to the standard deviation in the x-direction and the radius in the y-direction corresponds to the standard deviation in the y-direction.

None of the following graphs contain a legend. This is because the measured quantities are represented with colors that can be clearly assigned. One color can represent either the position of an apriltag (in Fig. 2 and 3) or the a quantity of an actuator, that is, the pressure p or bending angle α. The color scheme is as follows:

color	tag position	actuator
red	front left foot	front left leg
dark red	front right foot	front right leg
orange	front end of torso	left torso
dark orange	back end of torso	right torso
blue	rear left foot	rear left leg
dark blue	rear right foot	rear right leg

The orientation angle of the robot with respect to the global x-axis ε is represented in purple.

Each of the two versions climbed different inclinations – once with constant reference pressures and once with recalibrated reference pressures. This results in a total of four experiments. The following table links the figures to the corresponding experiment.

	large version	small version
constant pressure references	Figures 2, 4, 5	Figures 2, 8
recalibrated pressure references	Figures 3, 6, 7	Figures 3, 9

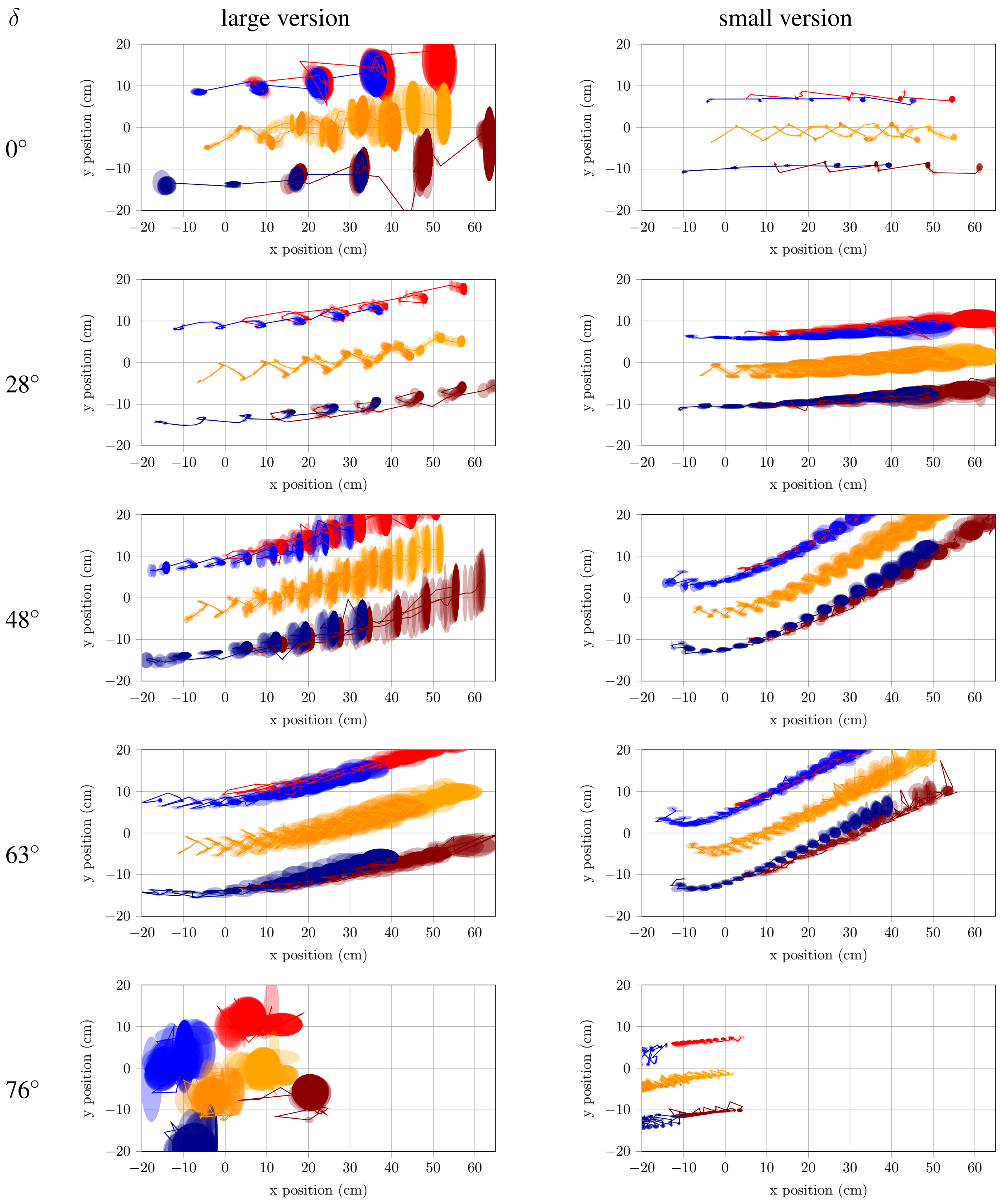

Figure 2. Track of feet of large (left column) and small (right column) prototype for different inclination angles δ, while performing straight gait. In this experiment the reference pressures are **not** recalibrated for each inclination.

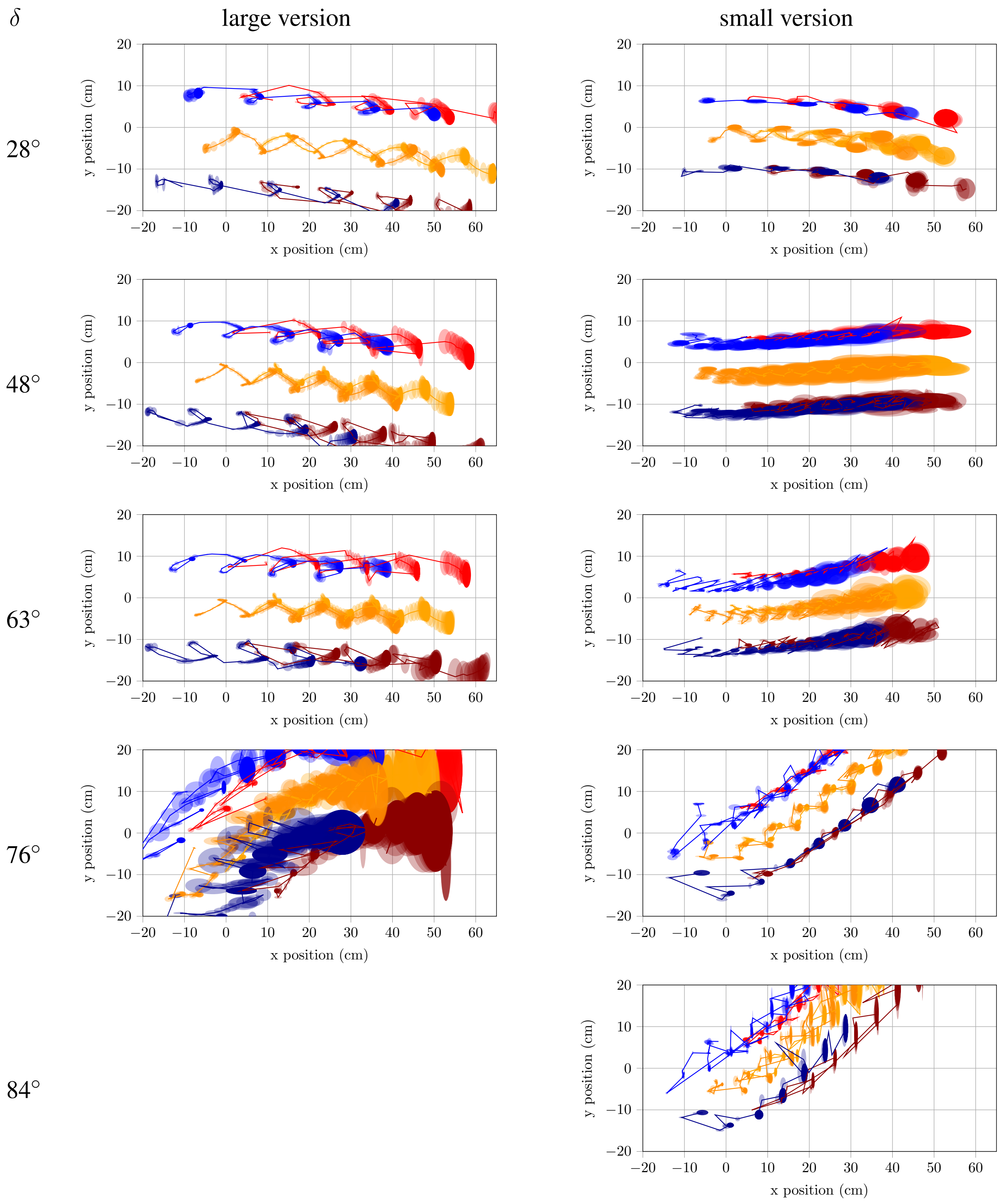

Figure 3. Track of feet of large (left column) and small (right column) prototype for different inclination angles δ, while performing straight gait. In this experiment the reference pressures are recalibrated for each inclination.

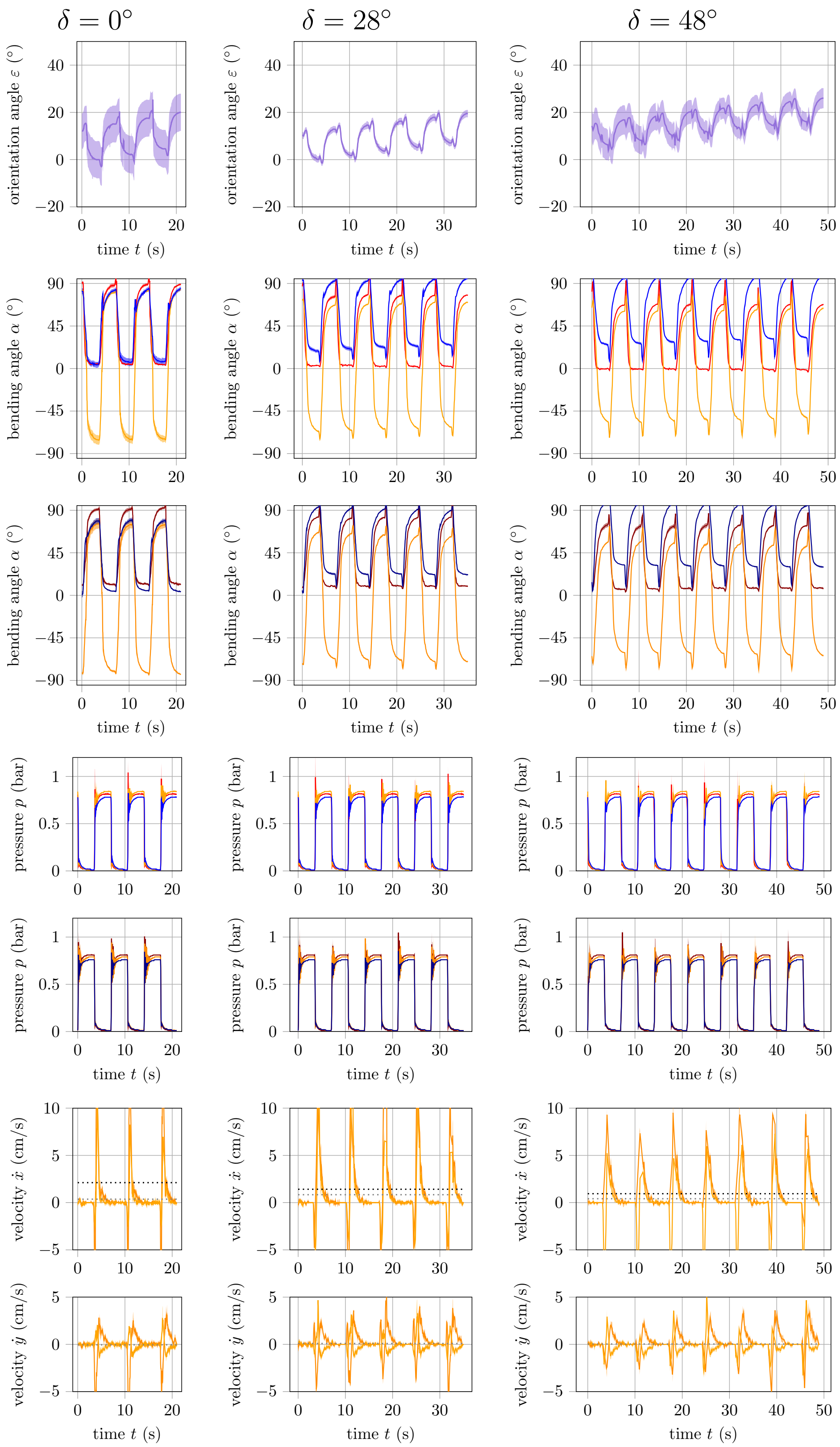

Figure 4. Measured data of large prototype for experiment with same pressure references for each inclination. Data for inclination 0°, 28°, 48°.

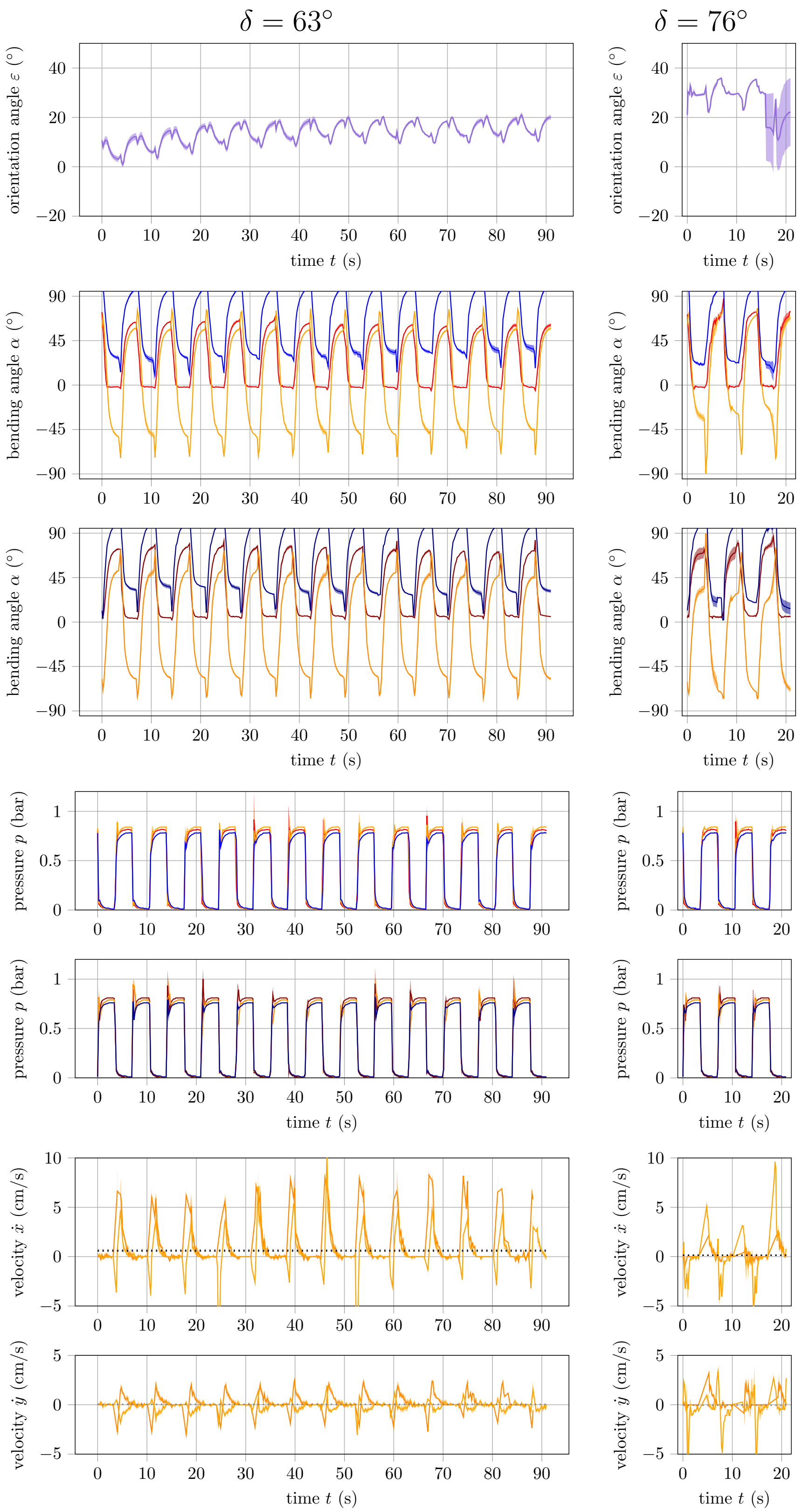

Figure 5. Measured data of large prototype for experiment with same pressure references for each inclination. Data for inclination 63°, 76°.

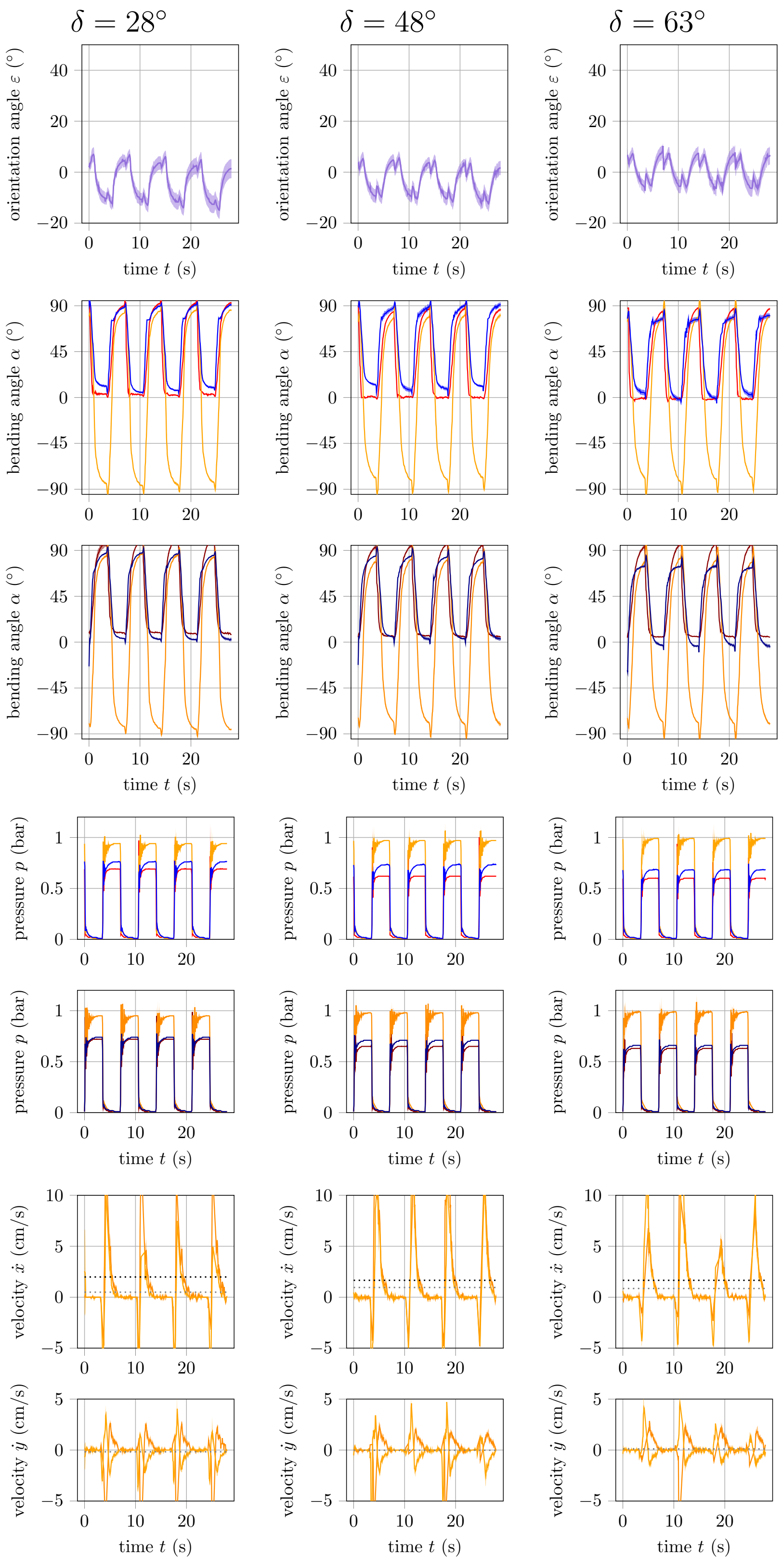

Figure 6. Measured data of large prototype for experiment with recalibrated pressure references for each inclination. Data for inclination 0°, 28°, 48°, 63°.

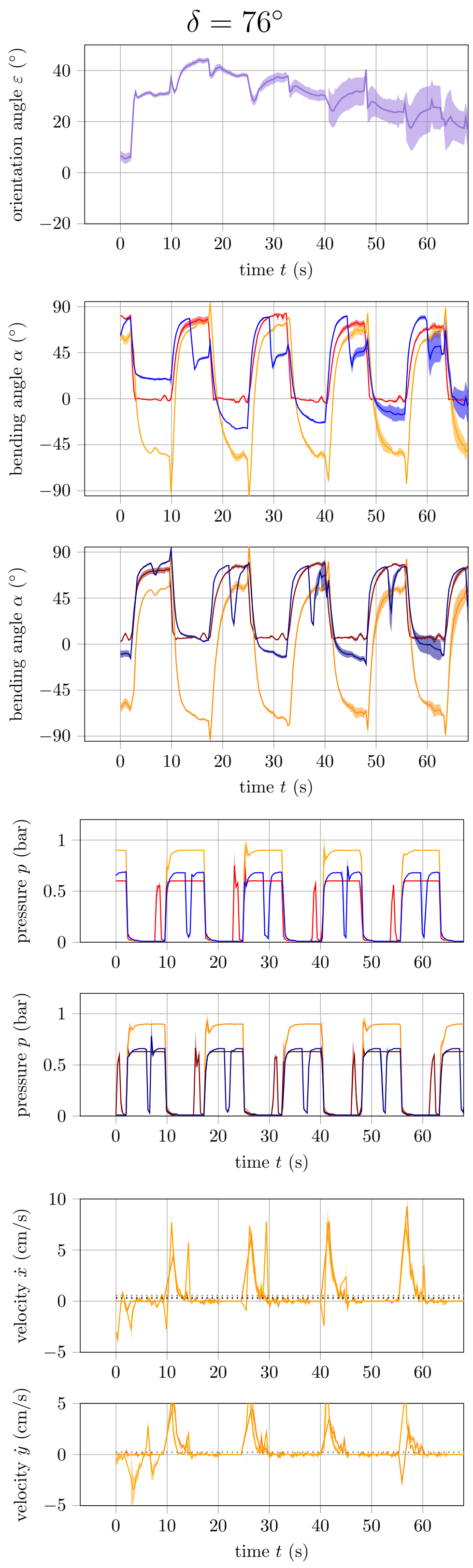

Figure 7. Measured data of large prototype for experiment with recalibrated pressure references for each inclination. Data for inclination 76°.

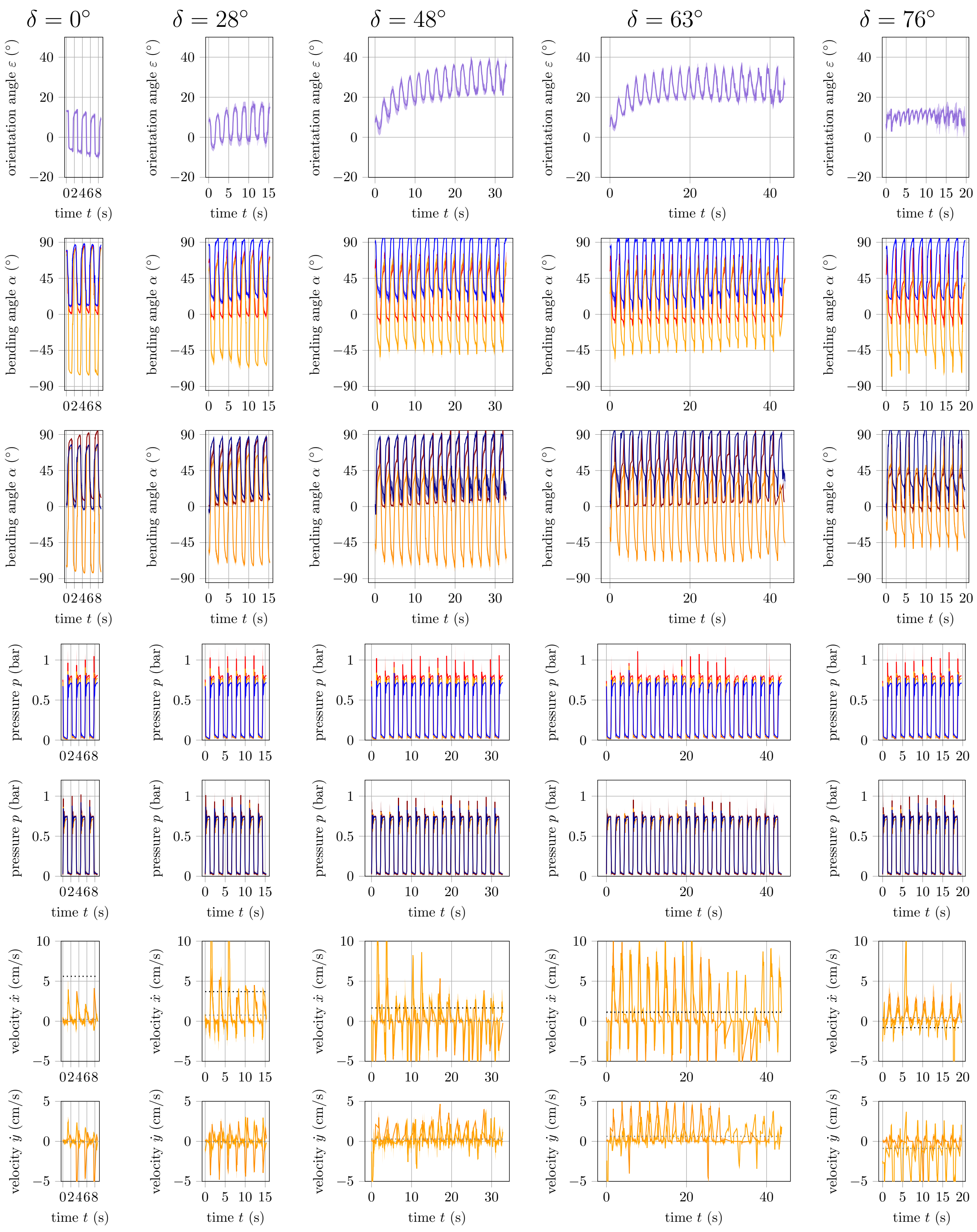

Figure 8. Measured data of small prototype for experiment with constant pressure references for each inclination.

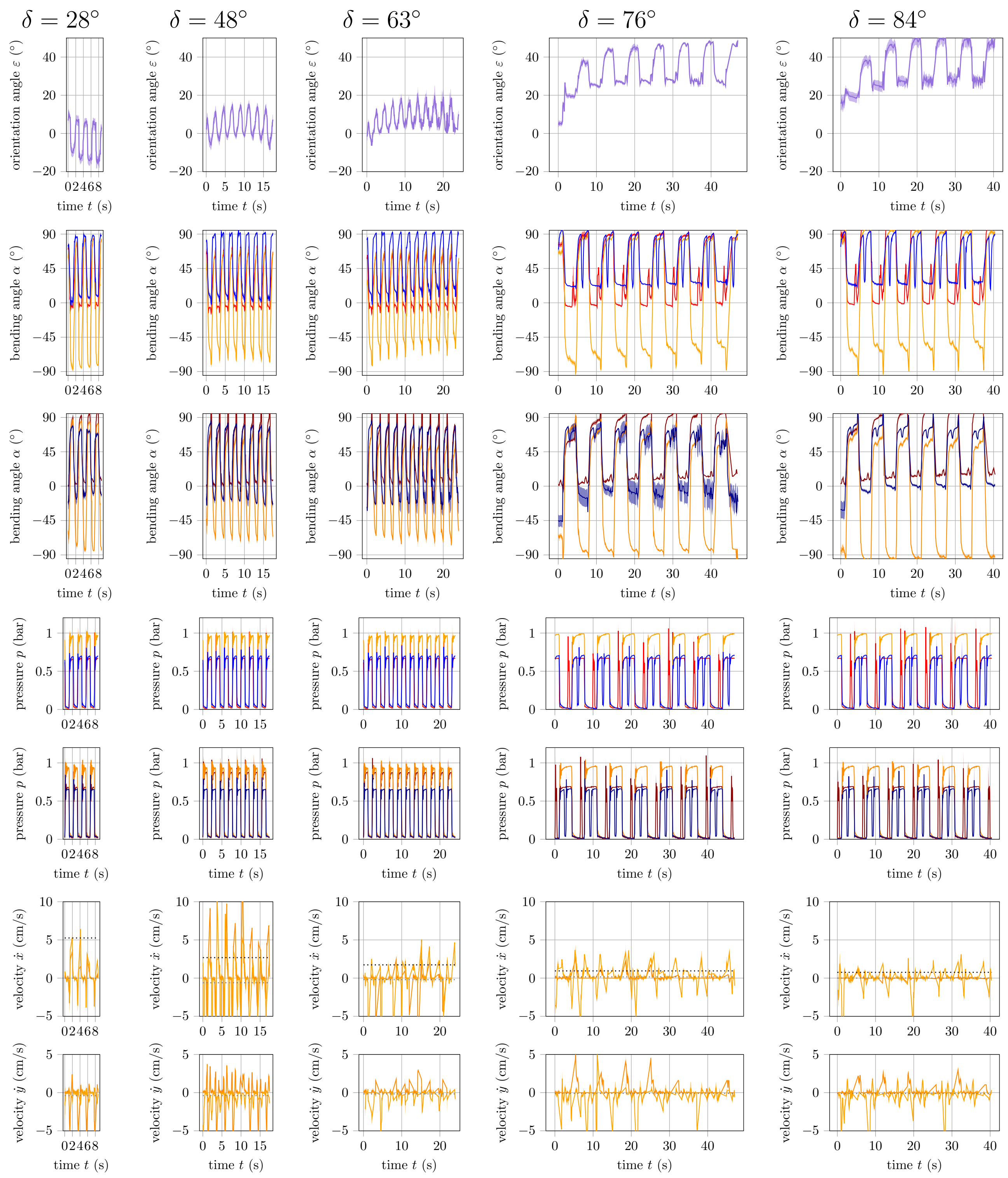

Figure 9. Measured data of small prototype for experiment with recalibrated pressure references for each inclination.
